# Stroma Cell-Derived Factor-1α Signaling Enhances Calcium Transients and Beating Frequency in Rat Neonatal Cardiomyocytes

**DOI:** 10.1371/journal.pone.0056007

**Published:** 2013-02-27

**Authors:** Ielham Hadad, Alex Veithen, Jean–Yves Springael, Panagiota A. Sotiropoulou, Agnès Mendes Da Costa, Françoise Miot, Robert Naeije, Xavier De Deken, Kathleen Mc Entee

**Affiliations:** 1 Department of Physiopathology, Faculty of Medicine, Université Libre de Bruxelles, Brussels, Belgium; 2 Institut de Recherche Interdisciplinaire en Biologie Humaine et Moleculaire, Faculty of Medicine, Université Libre de Bruxelles, Brussels, Belgium; 3 Chemcom S.A., Brussels, Belgium; University of Cincinnati, United States of America

## Abstract

Stroma cell-derived factor-1α (SDF-1α) is a cardioprotective chemokine, acting through its G-protein coupled receptor CXCR4. In experimental acute myocardial infarction, administration of SDF-1α induces an early improvement of systolic function which is difficult to explain solely by an anti-apoptotic and angiogenic effect. We wondered whether SDF-1α signaling might have direct effects on calcium transients and beating frequency.

Primary rat neonatal cardiomyocytes were culture-expanded and characterized by immunofluorescence staining. Calcium sparks were studied by fluorescence microscopy after calcium loading with the Fluo-4 acetoxymethyl ester sensor. The cardiomyocyte enriched cellular suspension expressed troponin I and CXCR4 but was vimentin negative. Addition of SDF-1α in the medium increased cytoplasmic calcium release. The calcium response was completely abolished by using a neutralizing anti-CXCR4 antibody and partially suppressed and delayed by preincubation with an inositol triphosphate receptor (IP_3_R) blocker, but not with a ryanodine receptor (RyR) antagonist. Calcium fluxes induced by caffeine, a RyR agonist, were decreased by an IP_3_R blocker. Treatment with forskolin or SDF-1α increased cardiomyocyte beating frequency and their effects were additive. *In vivo*, treatment with SDF-1α increased left ventricular dP/dtmax.

These results suggest that in rat neonatal cardiomyocytes, the SDF-1α/CXCR4 signaling increases calcium transients in an IP_3_-gated fashion leading to a positive chronotropic and inotropic effect.

## Introduction

The chemokine SDF-1α (Stroma cell-Derived Factor 1α) regulates a variety of cellular processes including cell homing and differentiation through its receptor CXCR4 [1]. The SDF-1α axis was shown to be activated in myocardial infarction. SDF-1α is over-expressed in ischemic myocardial tissue [2]–[4] and its observed cardioprotective effects are reported to be exerted through inhibition of cardiomyocyte apoptosis, stem cell recruitment and promotion of angiogenesis [5]. In rodent models of myocardial ischemia, SDF-1α administration has been shown to be associated with an early improvement in ventricular systolic function, which is difficult to explain solely on the basis of improved microcirculation and tissue preservation [5], [6]. We therefore wondered whether SDF-1α signaling might have intrinsic inotropic effects.

Calcium, by its rapid, non-genomic excitation-contraction coupling effect, is a key regulator of cardiac contractility and rhythm [7]. Calcium is also implicated in cardiac remodeling by gene transcription stimulation of hypertrophy mediators [8]. In adult cardiomyocytes, the dominant mode of intracellular calcium release is gated by ryanodine receptors (RyRs). This process depends on postnatal t-tubules formation and resultant colocalization of dihydropyridine receptors and RyRs [9]. On the other hand, during embryogenesis, inositol triphosphate-gated calcium release channels (IP_3_Rs) are important and in early postnatal development, an intermediate stage of calcium signaling is present, IP_3_Rs-gated calcium release being sufficiently large to contribute significantly to activation of calcium induced-calcium release by RyRs [10]. In human end-stage heart failure, RyRs are downregulated and IP_3_Rs are upregulated [11]. IP_3_Rs are also increased in animal models of hypertrophic myocardium and in human patients with ischemic dilated cardiomyopathy [12]. In heart failure, IP_3_Rs provide an alternative pathway for mobilizing intracellular calcium [11].

The majority of the SDF-1α mediated biological effects are initiated by binding to its cognate G-protein coupled receptor CXCR4. CXCR4, as many other chemokine receptors, is primarily coupled to the Gαi class proteins provoking adenylyl cyclase inhibition, as well as MAP-kinase and phosphatidylinositol 3-kinase pathways activation. The Gβγ subunit triggers phospholipase C activation and formation of IP_3_ resulting in mobilization of calcium from intracellular stores [13], [14]. In addition, CXCR4 can couple to other Gα proteins such as Gαq, αo and αs [15].

In the present study, we explored the effects of the interaction of SDF-1α with its receptor CXCR4 on neonatal cardiomyocyte calcium transients, in the presence or not, of either RyR or IP_3_R blockers. We have demonstrated that SDF-1α/CXCR4 signaling cascade increases IP_3_-mediated calcium transients associated with positive chronotropic effects.

## Materials and Methods

This study was approved by the Institution ethical committee for animal research and all animals received human care in compliance with the “Guide for the Care and Use of Laboratory Animals” (http://www.nap.edu/catalog/5140.html).

### Primary culture of rat neonatal cardiomyocytes

For isolation and culture of rat neonatal cardiomyocytes, we used the protocol described by Harary et al [16] modified by Chlopčíková [17]. Whole hearts from 2- to 5-day old rats were isolated, minced and rinsed in a balanced salt solution containing: CaCl_2_ 1.26 mM, NaCl 137 mM, NaH_2_PO_4_ 0.338 mM, MgSO_4_ 5.5 mM, KCl 5.3 mM, MgSO_4_ 0.8 mM, KH_2_PO_4_ 0.44 mM, NaHCO_3_ 4.17 mM, at pH 7.3–7.4. Five cycles of digestion using collagenase (95 U/ml) (C0130, Sigma Aldrich, Bornem, Belgium) and 0.6 U/ml of pancreatin (P7545, Sigma Aldrich) were performed. During each round of digestion, the tissue pieces were incubated and shaken gently for 20 min at 37°C in the balanced salt solution. At the end of each cycle, the suspension was centrifuged (500 g, 10 min), the supernatant collected, kept on ice and the cell pellet resuspended in 4 ml of the digestion solution. Supernatants from the 5 cycles were pooled, centrifuged (500 g, 10 min) and resuspended in the cardiac medium containing DMEM and M199 (4∶1) supplemented with horse serum (10%), fetal calf serum (5%), penicillin (100 U/ml) and streptomycin (100 mg/ml). After cell plating on non-coated culture dishes for 2 h to allow differential attachment of non-myocyte cells, the remaining cell suspension containing neonatal cardiomyocytes was collected, counted, and seeded at 5.10^4^ cells/cm^2^ in collagen I-coated culture dishes in the cardiac medium and incubated in 5% CO_2_ at 37°C. The medium was replaced after 72 h and cardiomyocytes were allowed to reach confluence before use. The original adherent non-myocyte enriched fraction was cultured under the same conditions and used as negative controls.

### Immunofluorescence analysis

For immunostaining, cells were plated on gelatin-coated slides, fixed for 20 min at room temperature with a phosphate buffer saline (PBS) solution containing 4% (w/v) paraformaldehyde and incubated with a mouse anti-troponin I (1∶100) (clone 13-11, NeoMarkers, Fremont CA, USA) and a rabbit anti-vimentin (1∶100) (D21H3, BIOKE, Leiden, The Netherlands) or with a mouse anti-CXCR4 (1∶100) (ABIN 223598, antibodies-online GmbH, Aachen, Germany) in PBS containing BSA 1%, Triton X-100 0.2% and donkey serum 5%. Primary antibodies were revealed with the respective anti-mouse Alexa Fluor 488 secondary antibody (1∶200) (F14201, Life Technologies, Ghent, Belgium) for troponin I and CXCR4 and anti-rabbit Rhodamine-Red X (RRX) (1∶400) (R 711-295-152, Jackson laboratories West Grove, PA, USA) for vimentin then counterstained with DAPI. Images were collected with a Zeiss Axio fluorescence microscope equipped with a camera and analyzed using the Axiovision Rel.4.6 software. Non specific staining was assessed by omission of the primary antibody. Staining was performed in triplicate, using samples from different culture preparations. Quantification was made by two blinded observers.

### Quantitative real time PCR (QRT-PCR)

Total RNA was extracted using the TRIzol reagent (Life Technologies, Ghent, Belgium) followed by a chloroform/ethanol extraction and an additional purification on columns (RNeasy kit, Qiagen, Venlo, Netherlands). RNA was quantified by spectrophotometry (NanoDrop ND-1000, Nanodrop technologies, Wilmington, USA). The quality was checked using the Experion automated electrophoresis method (Bio-Rad, Hemel Hempstead, UK). After first strand cDNA synthesis of 1 µg RNA, Sybr Green QRT-PCR was performed with the Icycler (Bio-Rad Laboratory, Nazareth, Belgium). Primers were designed to recognize rat cDNA sequences of GAPDH (used as a housekeeping gene), IP_3_R, CXCR4 and RyR (Table S1). For each primer, the PCR conditions were optimized to obtain only the specific product with an efficiency calculated from dilution curves between 95 and 105%. Each sample was measured in triplicate and each plate contained negative and positive controls. Melt curves were produced at the end of each plate processing. Relative RNA expression for each transcript of interest was analyzed using the Pfaffl method.

### Calcium imaging assay

Cells were seeded on poly-D-lysine pre-coated 96-well plates (343-2035, BD Biosciences, Erembodegem-Dorp, Belgium) at 2.10^4^ cells per well and incubated with the cardiac medium. To determine whether SDF-1α triggers calcium fluxes, 24 h post-seeding cells were washed with the calcium assay buffer containing: NaCl 150 mM, KCl 5 mM, CaCl_2_ 2 mM, MgCl_2_ 12 mM, glucose 10 mM, HEPES 10 mM, 0.01% (v/v) Pluronic F127 and 0.1% (w/v) BSA and loaded with 1 µg/ml of Fluo-4 acetoxymethyl ester (Fluo-4 AM) (F14201, Life Technologies, Ghent, Belgium). After 1 h of incubation at 37°C in darkness, cells were rinsed twice with the buffer and kept in 50 µl of buffer per well until measurements. Analyses were performed on a platform constituted by a motorized Axiovert 200 fluorescence microscope piloted by the KS400 software (Zeiss, Jena, Germany) and coupled with an EDOS electronic pipette (Eppendorf, Hamburg, Germany) used as injector. Macrorunning under KS400 that allows repetitive and automated injection and time lapse recording were developed by Chemcom S.A. (Brussels, Belgium). Calcium fluctuation was recorded within a total time course of 120 s by taking one picture per second. Fifty µl buffer containing the tested drug at twice the final concentration were injected in the well after a delay of 5 s that allows the determination of the calcium basal level. Drugs tested were the following: 0.05 µM, 0.5 µM,1 µM, 5 µM and 10 µM SDF-1α (350-NS, R&DSystems, Oxon, United Kingdom), 10 µM ATP (A64.19, Sigma Aldrich), 10 mM caffeine (27.602, Sigma Aldrich), 0.5 mg/ml anti-CXCR4 (5540-100, Biovision Imtec, Antwerp, Belgium), 1 mM tetracaine (T38.12, Sigma Aldrich), 2 µM 2-aminoethyl-diphenyl-borinate (2-APB) (D9754, Sigma Aldrich) and 10 µM forskolin (F1026, A.G. Scientific, Inc., San Diego, CA, USA). Excitation was set at 488 nm and emission monitored at 530 nm. All experiments were carried out at room temperature. The calcium signal was expressed as the maximal fractional change in whole field fluorescence light intensity: ΔF/F_0_ = (Fmax-F_0_)/F_0_, where F_0_ is the mean value of emitted fluorescent light in the selected field before drug application and Fmax is the peak of fluorescence light intensity of the same field after drug application [10]. Time to peak (seconds) was calculated as the time required for the transient calcium signal to reach the ΔF/F_0_ [18].

The Image.J 1.34 imaging software (Rasband, W.S., ImageJ, NIH, Bethesda, Maryland, USA, http://rsb.info.nih.gov/ij/) was used for all fluorescence analyses.

### Cell contraction frequency assay

Cardiomyocytes were seeded on poly-D-lysine-coated 96 well plates. Spontaneously contracting cell monolayers were treated with the appropriate concentration of agonist or buffer in serum-free DMEM without phenol red. Images of contracting cells were recorded on a Zeiss Axio Imager equipped with a Zeiss Axiocam Men camera and using the Axiovision Rel.4.6 software. Cell beating frequency was quantified by counting the number of monolayer contractions per minute before and after stimulation and the ratio of beating frequency after/before stimulation was calculated.

Forskolin, known to increase the beating frequency, was tested in a concentration-response protocol. The additive effect of SDF-1α on forskolin-induced increase of beating frequency was also investigated.

### Caspase-3 activity

Briefly, 20,000 cells per well were seeded in 96 well culture plates and cultured in serum-free medium in the presence or absence of SDF-1α (5 µM). Apoptosis was induced by hypoxia (1% O_2_ for 24 h) and/or staurosporine (ST) 1 µM (S4400, Sigma, St Louis MO, USA**)** for 8 h with or without a caspase-3 inhibitor (CASP3I) (CASP3F, Sigma, St Louis MO,USA). The caspase-3 activity was evaluated using a fluorimetric commercial kit (CASP3F, Sigma, St Louis MO, USA) according to manufacturer's instructions.

Data are expressed as fold change in caspase-3 activity fluorescence (F) compared to the control normoxic condition (F0).

### *In vivo* study

Eight male Wistar rats (250 g to 350 g) were anesthetized with isoflurane (1- 2%), orally intubated and mechanically ventilated. A Mikro-Tip 2F pressure catheter (Millar Instruments, Houston, Texas, USA) was introduced through the right carotid artery into the left ventricle. A left lateral thoracotomy was performed. After opening the pericardium, 100 µl of SDF-1α reconstituted at a concentration of 100 ng/µl in sterile PBS containing 0.1% BSA (treated, n = 4), or 100 µl of PBS +0.1% BSA (placebo, n = 4) was delivered into the left ventricular posterior wall in four sites using a 29G needle. Left ventricular systolic pressure (LVSP), maximal rate of rise in left ventricular pressure (dP/dtmax) and heart rate were recorded with a commercial software (IOX; Emka Technologies SA, Paris, France) after injection and again 5, 10 and 15 minutes later.

### Statistics

Results are expressed as mean ± SEM. The normality of distribution was tested with a Shapiro-Wilk test (Sigma stat Software). For normally distributed data we used Student's t-test. When the Shapiro-Wilk test failed, differences within the groups were analyzed by a rank sum test. *In vivo* variables were tested by a two-factor analysis of variance (ANOVA) for repeated measures followed by Scheffe post-hoc tests when overall significance was detected. Differences were considered statistically significant when p<0.05.

## Results

### Phenotypic characterization of rat neonatal cardiomyocyte primary culture

#### Immunocytology

**Figure 1:**
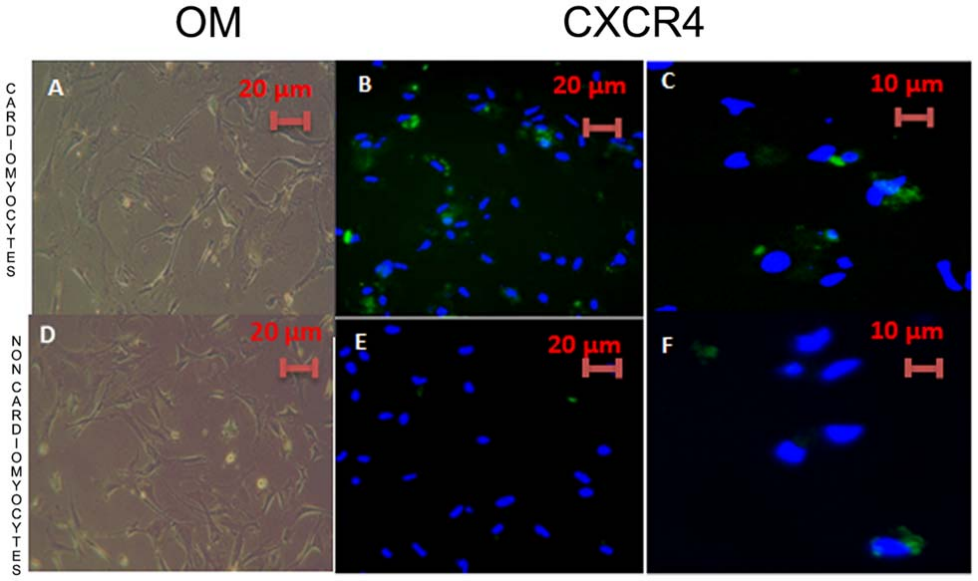
The cultured cardiomyocytes were immunostained in green for CXCR4 and counterstained with DAPI for nuclei (blue). The cardiomyocyte enriched fraction showed increased expressions of CXCR4 (B and C) compared to the non-myocyte enriched fraction (E and F). OM: Bright field for CXCR4 (A: cardiomyocyte enriched fraction, D: non-myocyte enriched fraction).

**Figure 2:**
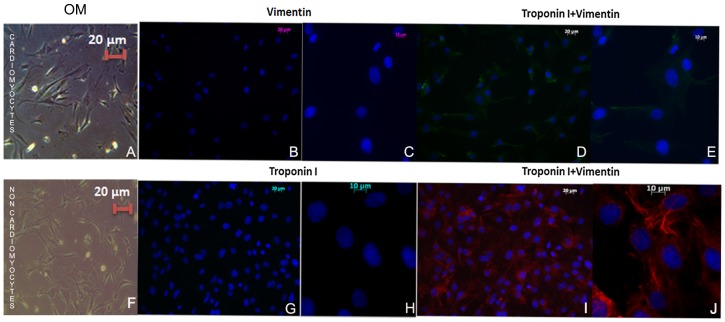
Cardiomyocyte and non-myocyte enriched fractions co-immunstained for troponin I (green) to mark myocytes, vimentin (red) to mark fibroblasts and with DAPI (blue) to mark the nuclei. The cardiomyocyte enriched fraction was troponin I positive (D and E) and vimentin negative (B–E) and the non-myocyte enriched fraction was vimentin positive (I and J) but troponin negative (G and H).

The cardiomyocyte enriched fraction formed a confluent monolayer of rod-shaped cells, some of which were beating spontaneously after 24 hours (Fig. S1A) while the non-myocyte enriched fraction presented a fibroblastic like appearance (Fig. S1B). CXCR4 was more frequently detected in neonatal cardiomyocytes (61±5%) (Fig. 1: B and C) than in the non-myocyte enriched fraction (37±4%, p<0.05) (Fig. 1: E and F). Troponin I was immunodetected in the majority of cells of the cardiomyocyte enriched fraction (98±1%) (Fig. 2: D and E) while only 2±1% of the cells of the non-myocyte enriched fraction was troponin I positive (Fig. 2: G–J). Vimentin was immunodetected in the majority of the non-myocyte enriched fraction (98±1%) (Fig. 2: I and J) while the cardiomyocyte enriched fraction was vimentin negative (Fig. 2: B–E).

#### QRT-PCR

**Figure 3:**
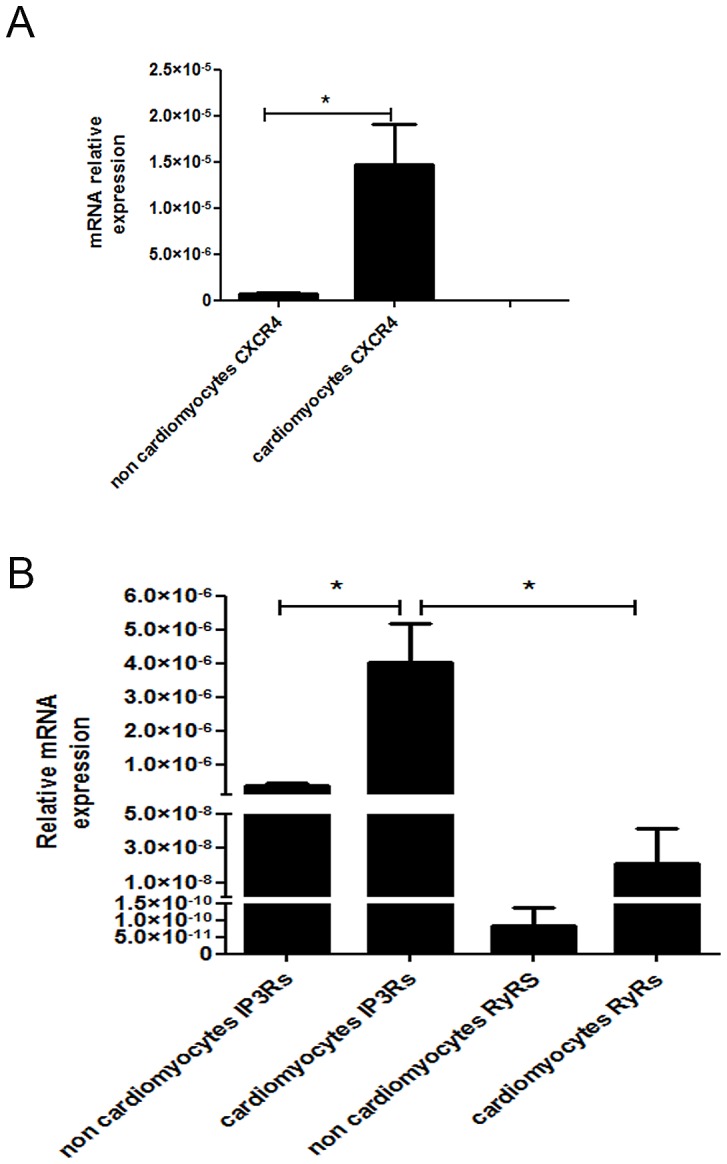
Relative gene expression of (A) CXCR4 and (B) IP_3_Rs and RyRs measured by QRT-PCR in the cardiomyocyte enriched fraction compared to the non-myocyte enriched fraction (n = 3). Data are presented as mean ± SEM, *P<0.05.

CXCR4 gene expression was higher in the cardiomyocyte enriched fraction compared to the non-myocyte enriched fraction (Fig. 3A). The cardiomyocytes expressed significantly more IP_3_Rs compared to RyRs (Fig. 3B).

### Calcium imaging assay

#### SDF-1α increased cardiomyocyte calcium fluxes through CXCR4 signaling

**Figure 4:**
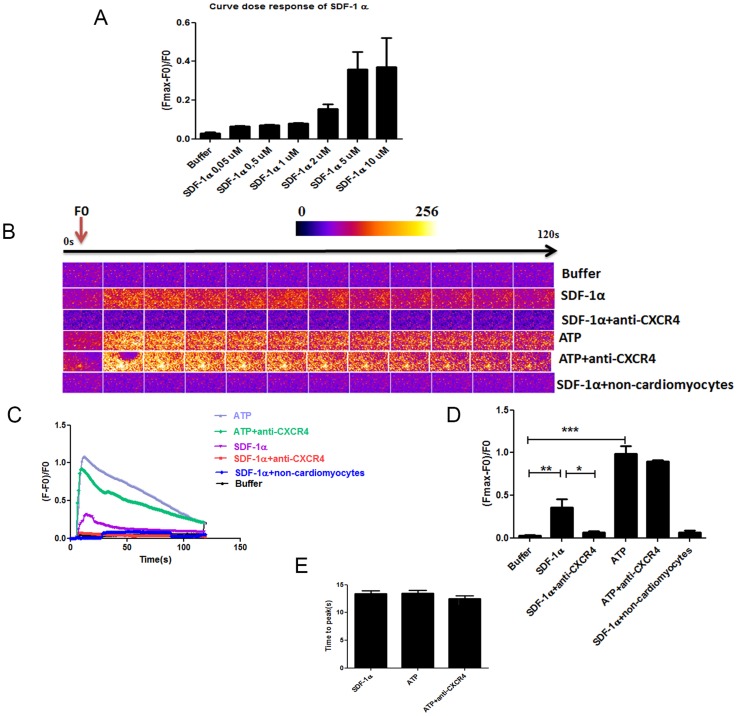
SDF-1α increased cardiomyocyte calcium transients through CXCR4. Ca^2+^ transients were evoked by 0.05 µM, 0.5 µM, 1 µM, 2 µM, 5 µM and 10 µM SDF-1α (n = 2), 5 µM SDF-1α 5 min after 0.5 mg/ml anti-CXCR4 preincubation (n = 4), 10 µM ATP (n = 5), ATP after 5 min anti-CXCR4 preincubation (n = 2) and finally 5 µM SDF-1α in the non-myocyte enriched fraction (n = 3). (A) Dose-response curve of the Ca^2+^ transient (Fmax-F_0_)/F_0_ induced by SDF-1α. (B, C) Time course of the Ca^2+^ fluorescent signal in a representative experiment. (D) Comparison of the Ca^2+^ transients (Fmax-F_0_)/F_0_ between groups. (E) Comparison of the time to peak. All data are presented as mean ± SEM, *P<0.05, **P<0.01, ***P<0.001.

The calcium signal induced by SDF-1α was tested at concentrations ranging from 0.05 to10 µM. A plateau was obtained at a concentration of 5 µM (Fig. 4A); this concentration was selected for subsequent experiments. Quantification of Fluo-4 fluorescence intensity (Fig. 4B) showed that the majority of the cells (75.0±3.7%) evidenced a calcium burst in response to SDF-1α injection whereas no significant calcium burst could be observed in cells (1.18±0.06%) after buffer injection. No calcium flux could be measured in the adherent non-myocyte enriched fraction treated with the drug (2.01±0.61%). The SDF-1α-mediated cytoplasmic calcium release in the cardiomyocytes (ΔF/F0 = 0.360±0.007 versus 0.029±0.007 after buffer, p<0.01) was almost completely abolished after a 1 h preincubation with an anti-CXCR4 antibody known to block CXCR4 activity (ΔF/F0 = 0.063±0.017, p<0.05 compared to SDF-1α alone), whereas the ATP-induced calcium response remained unaffected upon treatment with this neutralizing antibody (Fig. 4).

### The SDF-1α/CXCR4 axis increased cardiomyocyte calcium transients through activation of IP_3_Rs

**Figure 5:**
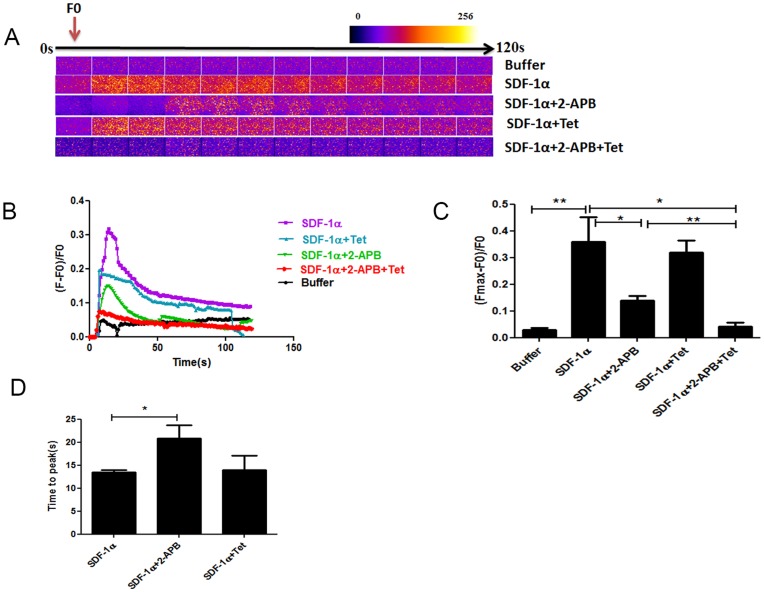
SDF-1α increased cardiomyocyte calcium transients through IP_3_R opening. Ca^2+^ transients were evoked by 5 µM SDF-1α (n = 5), SDF-1α 5 min after preincubation with 2 µM 2-APB (n = 4), SDF-1α 5 min after preincubation with 1 mM tetracaine (n = 3) and SDF-1α 5 min after preincubation with 2-APB + tetracaine (n = 4). (A, B) Time course of the Ca^2+^ fluorescent signal in a representative experiment. (C) Comparison of the Ca^2+^ transient (Fmax-F_0_)/F_0_ between groups. (D) Comparison of the time to peak. All data are presented as mean ± SEM, *P<0.05, **P<0.01. 2-APB: 2-aminoethyl-diphenyl-borinate, Tet: tetracaine.

To determine which intracellular cascade is involved in SDF-1α/CXCR4 mediated Ca^2+^ fluxes, we blocked separately or simultaneously different pathways involving IP_3_Rs or RyRs (Fig. 5).

Calcium fluxes induced by SDF-1α were partially suppressed (ΔF/F0 = 0.140±0.017, p<0.05 compared to SDF-1α alone) and delayed (20.8±2.84 sec versus 13.4±0.5, p<0.05) by preincubation with the 2-aminoethyl-diphenyl-borinate (2-APB), an IP_3_R blocker but not with tetracaine, an antagonist of RyRs (ΔF/F0 = 0.320±0.040, p = 0.72 compared to SDF-1α alone). Interestingly, simultaneous pretreatment with both 2-APB and tetracaine led to a complete inhibition of SDF-1α induced calcium burst (ΔF/F0 = 0.041±0.017, p<0.01 compared to SDF-1α alone).

### Activation of RyRs increased neonatal cardiomyocytes calcium transients

**Figure 6:**
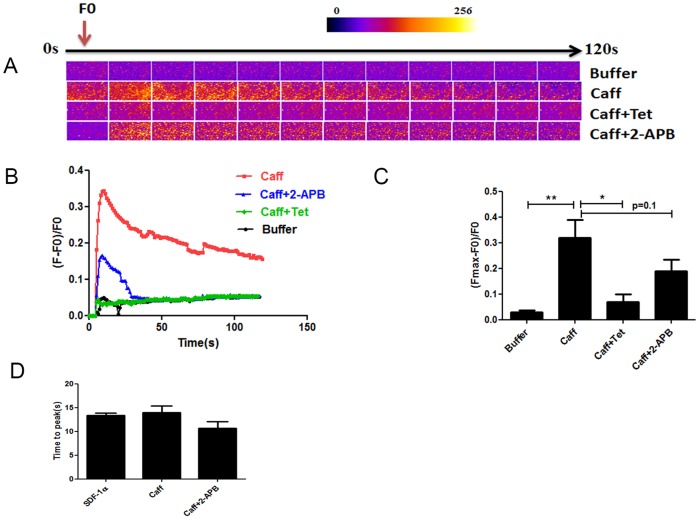
Activation of RyRs increased neonatal cardiomyocyte calcium transients. Ca^2+^ transients were evoked by 10 mM caffeine (n = 4), caffeine 5 min after preincubation with 1 mM tetracaine (n = 3) or 2 µM 2-APB (n = 3). (A, B) Time course of the Ca^2+^ fluorescent signal in a representative experiment. (C) Comparison of the Ca^2+^ transients (Fmax-F_0_)/F_0_ between groups.(D) Comparison of the time to peak. All data are presented as mean ± SEM, *P<0.05, **P<0.01. 2-APB: 2-aminoethyl-diphenyl-borinate, Tet: tetracaine, Caff: caffeine.

Caffeine, a RyR agonist, also enhanced calcium transients (ΔF/F0 = 0.320±0.060, p<0.01) compared to buffer. This response was abolished after treatment of the cells with tetracaine, (ΔF/F0 = 0.068±0.023, p<0.05 compared to caffeine alone) and tended to be reduced by pretreatment with the IP_3_R blocker (ΔF/F0 = 0.190±0.040, p = 0.1) (Fig. 6).

**Figure 7:**
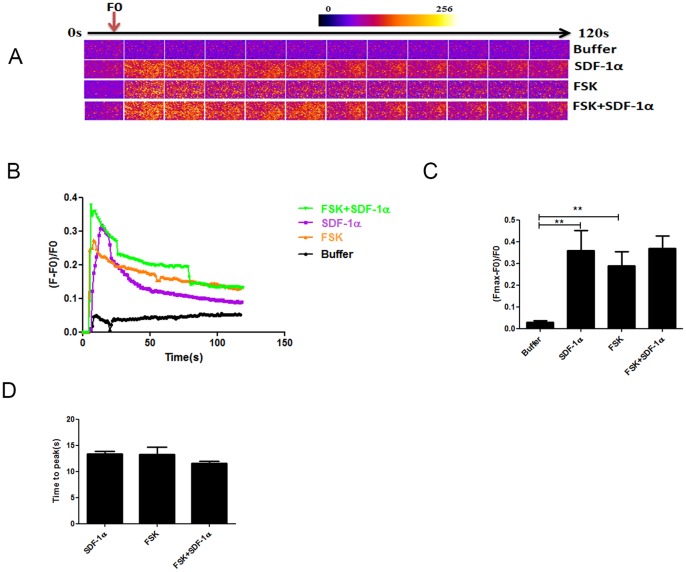
Forskolin induced calcium transients in neonatal cardiomyocytes. Ca^2+^ transients were evoked by application of 10 µM forskolin alone (n = 3) or in addition to 5 µM SDF-1α (n = 3). (A, B) Time course of the Ca^2+^ fluorescent signal in a representative experiment. (C) Comparison of the Ca^2+^ transient (Fmax-F_0_)/F_0_ between groups.(D) Comparison of the time to peak. All data are presented as mean ± SEM, **P<0.01. FSK: forskolin.

The cardiomyocytes were exposed to forskolin (10 µM), a direct activator of the adenylyl cyclase leading to a cAMP-mediated calcium release (Fig. 7). Forskolin increased calcium transients (ΔF/F0 = 0.290±0.026, p<0.05 compared to buffer) at the same level as with SDF-1α (p = 0.6 compared to forskolin) and their effects were not additive (ΔF/F0 = 0.370±0.056, p = 0.4 compared to forskolin alone).

### SDF-1α increased the beating frequency of the cardiomyocytes

**Figure 8:**
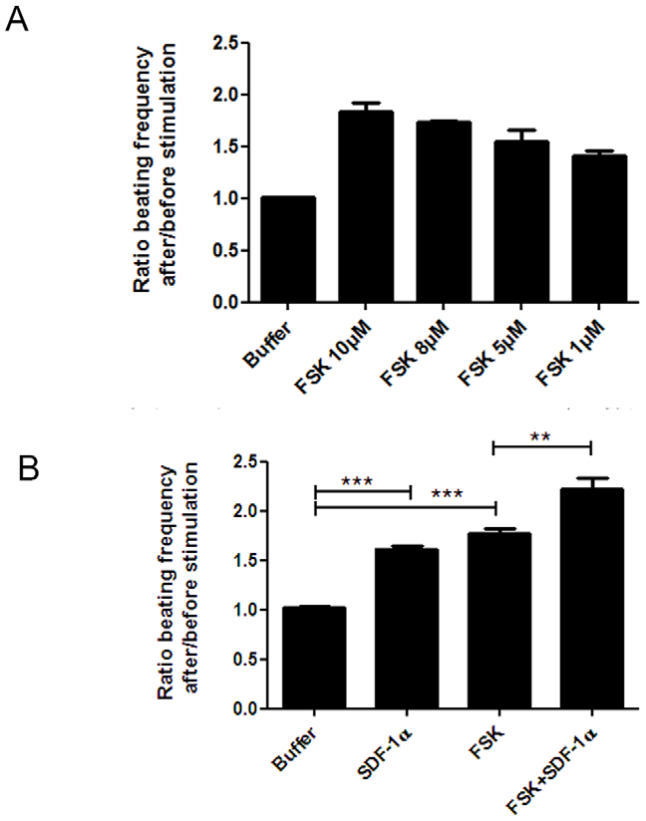
SDF-1α increased the cell beating frequency with additive effects to forskolin. (A) Concentration-response of forskolin on the beating frequency of spontaneous contracting cardiomyocytes presented as a ratio after/before stimulation. (B) 5 µM SDF-1α increased the beating frequency at the same level as forskolin. Forskolin (10 µM) and SDF-1α were additive in terms of their effect on the beating frequency (n = 6). All data are presented as mean ± SEM, **P<0.01, ***P<0.001. FSK: forskolin.

Forskolin increased the cardiomyocyte beating frequency in a concentration dependent manner with a maximum response observed at 10 µM (Fig. 8A, Video S2). Exposure of spontaneously contracting cardiomyocytes to SDF-1α (5 µM) increased the cell beating frequency (1.610±0.028, p<0.001 versus buffer) (Fig. 8B, Video S1). A significant additive effect of SDF-1α to forskolin (10 µM) (2.250±0.108 versus 1.760±0.047 for forskolin alone, p<0.01) (Fig. 8B, Video S4) could also be observed.

### Caspase-3 activity

**Figure 9:**
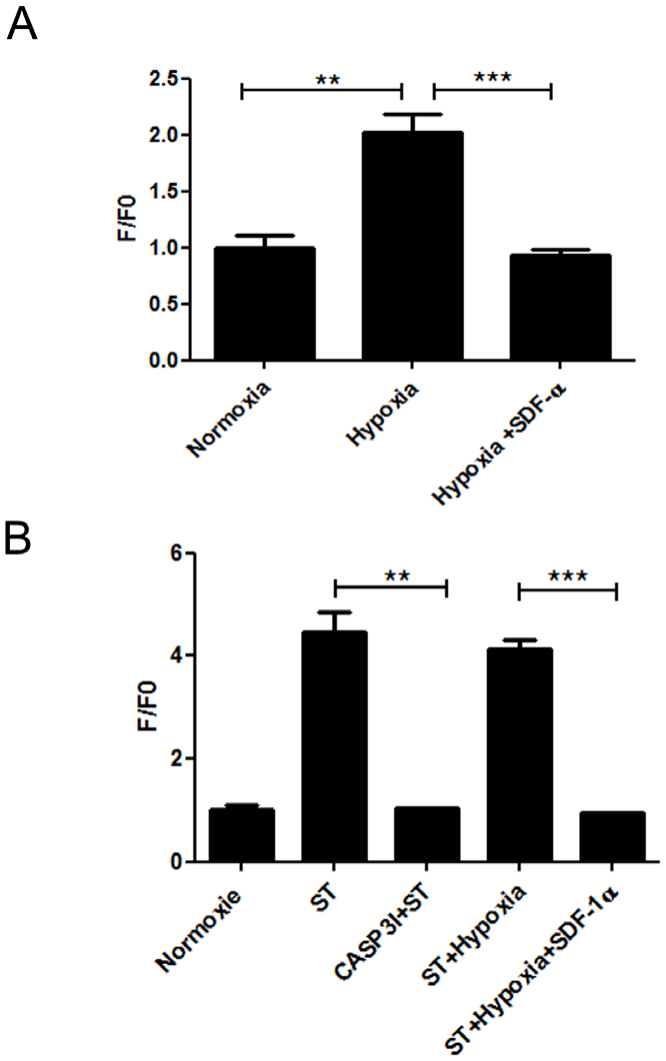
SDF-1α decreased apoptosis in neonate cardiomyocytes. (A) 5 µM SDF-1α reduced apoptosis induced by hypoxia (n = 4) compared to normoxia (n = 4). (B) 5 µM SDF-1α protected against apoptosis induced by hypoxia combined to staurosporine 1 µM (n = 3). All data are expressed as fold change in Caspase-3 activity fluorescence (F) compared to normoxia (F0) *P<0.05, **P<0.01, ***P<0.001. ST: staurosporine, CASP3I: Caspase-3 inhibitor.

To characterize the anti-apoptotic effect of SDF-1α on neonate cardiomyocytes, we measured the caspase-3 activity. A marked increase of caspase-3 activity was observed in hypoxia (2.01±0.15, p<0.01), staurosporine (4.41±0.38, p<0.001) and hypoxia + staurosporine (4.13±0.17, p<0.001) compared to the normoxia condition (0.99±0.11) (Fig. 9). Treatment with SDF-1α for 24 h abolished apoptosis induced by hypoxia (0.93±0.046, p<0.001) and by hypoxia combined to staurosporine (0.93±0.033, p<0.001) (Fig. 9B).

### *In vivo* study

#### SDF-1α increased cardiac systolic function

**Figure 10:**
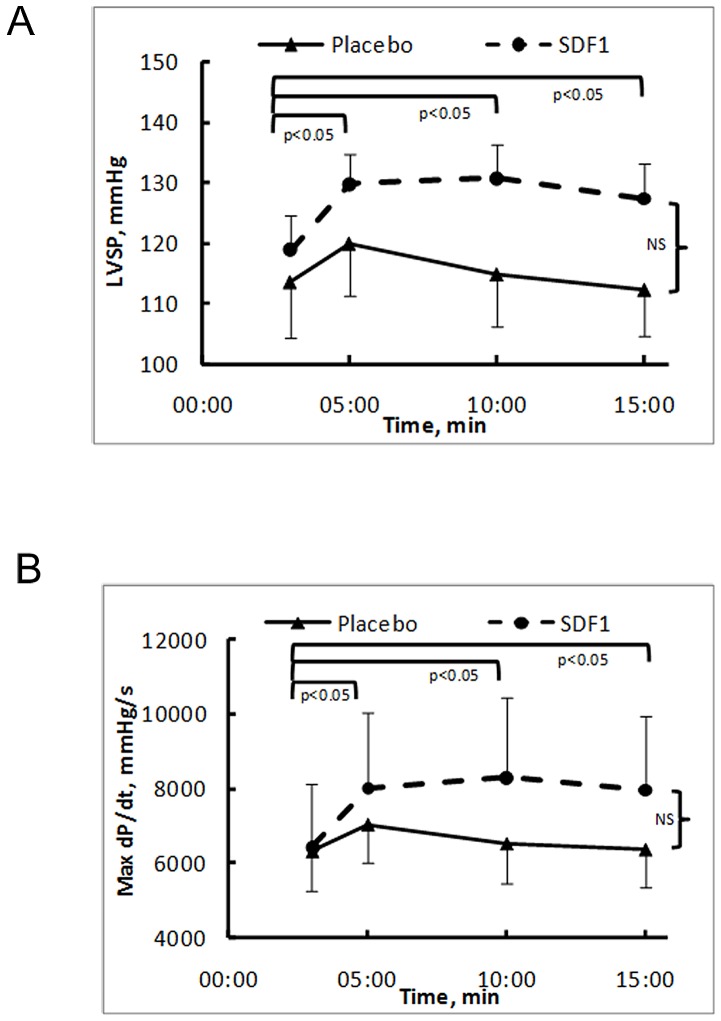
SDF-1α increased dP/dtmax *in vivo*. (A) Time-response of SDF-1α or placebo injection on left ventricular systolic pressure (LVSP) (n = 4 per group). (B) Time-response of SDF-1α or placebo injection on the maximal rate of rise in left ventricular pressure (dP/dtmax).

SDF-1α injected in the left ventricular posterior wall induced an increase in LVSP (Fig. 10A) and dP/dtmax (Fig. 10B) while LVSP and dP/dtmax stayed unchanged after injection of the same volume of SDF-1α solvent (placebo). No significant change in heart rate was observed.

## Discussion

The present results demonstrate that, besides its protective effect against cardiomyocyte apoptosis, SDF-1α/CXCR4 signaling increases cytosolic calcium transients in an IP_3_-dependent manner and induces a positive chronotropic effect in rat neonatal cardiomyocytes *in vitro* while it increases cardiac systolic function *in vivo*. These observations may offer an explanation for previously reported rapid improvements of ventricular systolic function in ischemic myocardium after the administration of SDF-1α.

Intramyocardial administration of SDF-1α, in mice with myocardial infarction produced by coronary artery ligation, has been shown to be associated with activation of the cell surviving factor protein kinase B, upregulation of the vascular endothelial growth factor, neo-angiogenesis and improvement of echocardiographic indices of ventricular systolic function such as fractional shortening and ejection fraction [6]. Intracoronary administration of SDF-1α in rats with experimental myocardial ischemia-reperfusion injury reproduces these cardioprotective and angiogenic effects in association with increased ejection fraction and decreased end-systolic and end-diastolic volumes measured invasively [5]. In both studies, indices of left ventricular systolic function were improved at the earliest non invasive (echocardiography, 24 h) or invasive (pressure-volume loops, 3 h) measurements [5], [6]. This is too early with respect to the time course of expressions of anti-apoptotic and angiogenic factors, so that additional mechanisms would have to be invoked to explain these SDF-1α-induced functional effects.

Cardiac contractility and rhythm are mainly determined by cytosolic calcium sparks from the sarcoplasmic reticulum leading to cardiomyocyte excitation-contraction coupling [7]. In healthy adult cardiomyocytes, calcium fluxes are essentially mediated by ryanodine receptors [9]. However, a significant contribution of IP_3_ receptors has been reported in failing or hypertrophied cardiomyocytes [11], [12] and has also been shown to be a feature of developing cardiomyocytes [10]. Accordingly, in the present study, we selected neonatal cardiomyocytes for the investigation of IP_3_ and ryanodine receptors mediated calcium transients as a model of failing cardiomyocytes. SDF-1α/CXCR4 signaling markedly increased IP_3_-gated calcium transients. The effect was specifically blocked using a neutralizing anti-CXCR4 antibody, reduced and delayed by an IP_3_ blocker but was insensitive to RyR blockade. SDF-1α mediated calcium release could be completely suppressed only in the simultaneous presence of the inhibitors against IP_3_Rs and RyRs. Moreover, caffeine-mediated calcium release tended to be reduced by inhibition of IP_3_Rs. These results confirm the role of two distinct calcium channels in neonatal cardiomyocytes namely an IP_3_-gated calcium release and a RyR calcium release and suggest a certain degree of cross-talk between these calcium channels.

*In vitro*, our data demonstrated that SDF-1α increased the beating frequency in the same range as the response to forskolin, a direct adenylyl cyclase activator. Moreover, *in vivo*, SDF-1α increased dP/dtmax, an index of cardiac systolic function leading to an increase in the left ventricular maximal pressure. These *in vitro and in vivo* results prove that the effects of SDF-1α on calcium cycling are functionally relevant. Moreover, forskolin and SDF-1α were additive in terms of their effect on beating frequency but, addition of SDF-1α to forskolin did not lead to any further changes in calcium transients. These observations suggest that another mechanism, independent of calcium, targeting directly the myofilaments might also participate to the observed functional chronotropic effect. These results are at variance with the negative inotropic effect of CXCR4 signaling demonstrated on rat papillary muscle and isolated cardiomyocytes [19]. In addition, a recent report showed that SDF-1α/CXCR4 signaling decreases β-adrenergic receptor-induced protein kinase A activity as assessed by cAMP accumulation and phosphorylation of phospholamban [20]. Both studies were performed on isolated adult rat cardiomyocytes in which excitation-contraction coupling is ryanodine rather than IP_3_-gated [9] while, in our model, gene expression was higher for IP_3_Rs than for RyRs and the two pathways contributed to mobilization of intracellular calcium.

Initial studies on the potential role of SDF-1α in cardiac regeneration focused on the homing of bone marrow-derived somatic stem cells to the heart after infarction [21]. Previous reports have suggested that some apparently tissue-specific progenitors may have differentiation potential outside their tissue of origin [22]. However, there has been doubt more recently about the potential of bone marrow-derived stem cells to differentiate into cardiomyocytes [23], although they may exert beneficial effect through secretion of paracrine growth factors [24]. Whether SDF-1α induced calcium transient increase, positive chronotropic and inotropic effects are beneficial after acute myocardial infarction remain unclear. Expression and activity of calcium cycling proteins are altered in heart failure causing less sarcoplasmic reticulum calcium uptake and release [25]. These changes play a critical role in contractile dysfunction and arrhythmogenesis [26]. These observations suggest that patients may benefit from an increase in sarcoplasmic reticulum calcium release. On the other hand, currently available inotropic drugs that act by increasing cytosolic calcium have consistently failed to show clinical benefit beyond short-term functional improvements in patients with heart failure [27]. Moreover, increased IP_3_Rs in cardiac hypertrophy may increase the propensity to arrhythmias [12].

In conclusion, the SDF-1α/CXCR4 axis increases calcium transients in an IP_3_-gated fashion in rat neonatal cardiomyocytes, leading to a positive chronotropic and inotropic effect. Our observations concern early improvements in ventricular systolic function reported after administration of SDF-1α to adult ischemic hearts. Whether this effect would be beneficial or not remains to be explored.

## Supporting Information

Figure S1 **The cardiomyocyte enriched fraction formed a confluent monolayer of rod-shaped cells (A) compared to the fibroblastic appearance of non-myocyte enriched fraction (B).**
(TIF)

Video S1 **Time lapse videos of a representative experiment showing that 5 µM SDF-1α (A) enhanced the beating frequency in neonatal cardiomyocytes.**
(ZIP)

Video S2 **Time lapse videos of a representative experiment showing that 10 µM forskolin (B) enhanced the beating frequency in neonatal cardiomyocytes.**
(ZIP)

Video S3 **Time lapse videos of a representative experiment showing that the buffer (C) didn't enhanced the beating frequency in neonatal cardiomyocytes.**
(ZIP)

Video S4 **Time lapse videos of a representative experiment showing that forskolin and SDF-1α showed additive effects (D).**
(ZIP)

Table S1 **Sequences of upstream and downstream oligonucleotide primers.**
(DOCX)
