# Supplementary figures and images for: Stroma Cell-Derived Factor-1α Signaling Enhances Calcium Transients and Beating Frequency in Rat Neonatal Cardiomyocytes
Source: PLoS One. 2013 Feb 27;8(2):e56007. doi: 10.1371/journal.pone.0056007 (PMC3584107; doi:10.1371/journal.pone.0056007)

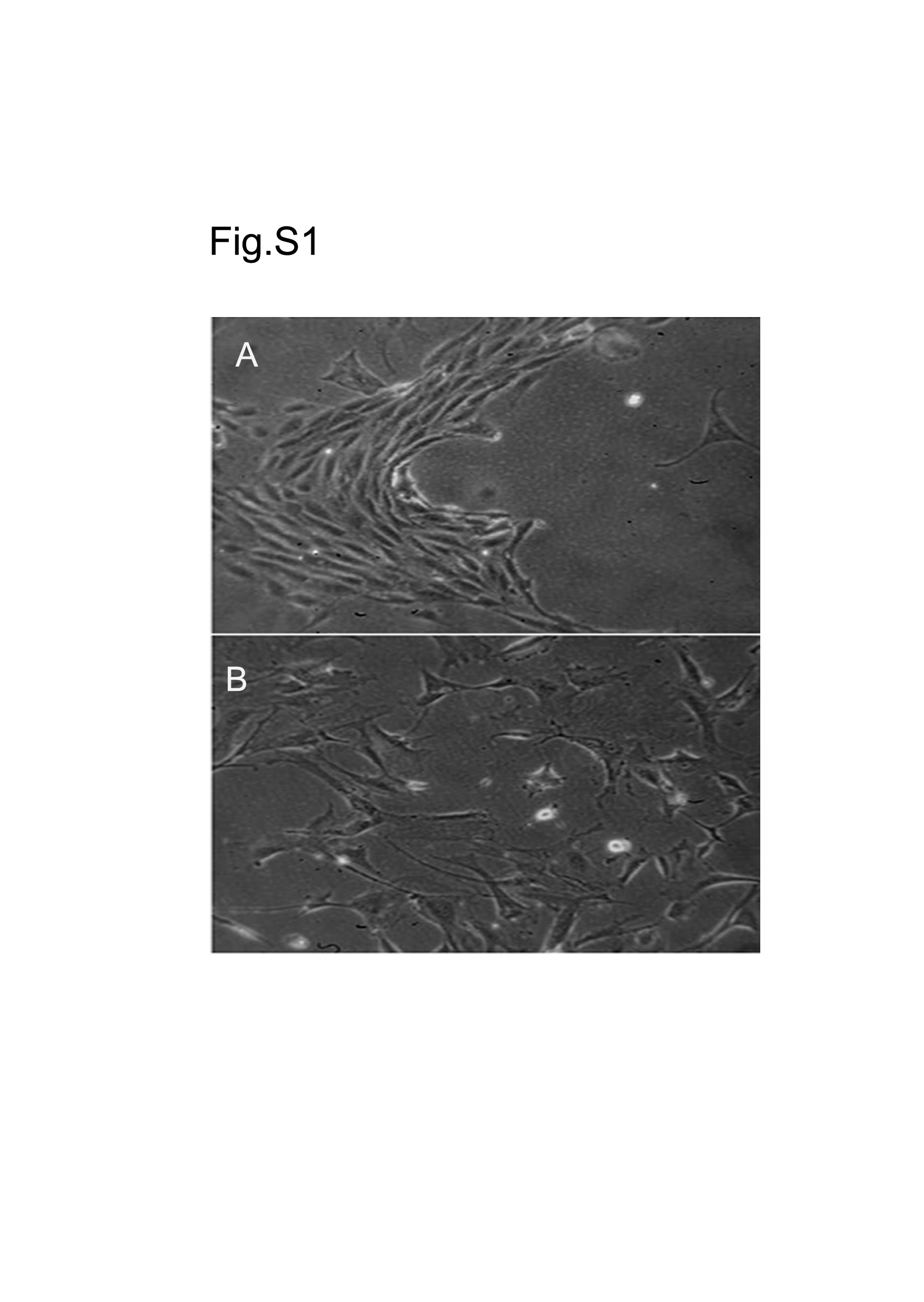

Supplement: Figure S1 — The cardiomyocyte enriched fraction formed a confluent monolayer of rod-shaped cells (A) compared to the fibroblastic appearance of non-myocyte enriched fraction (B). (TIF) [file pone.0056007.s001.tif]
