# Supplementary material for: Stroma Cell-Derived Factor-1α Signaling Enhances Calcium Transients and Beating Frequency in Rat Neonatal Cardiomyocytes
Source: PLoS One. 2013 Feb 27;8(2):e56007. doi: 10.1371/journal.pone.0056007 (PMC3584107; doi:10.1371/journal.pone.0056007)
Supplement: Table S1 — Sequences of upstream and downstream oligonucleotide primers. (DOCX) [file pone.0056007.s006.docx]

**Table S1**: Sequences of upstream and downstream oligonucleotide primers

| **Gene** | **Primers sequences** |
| --- | --- |
| **GAPDH**  Sense  Antisense | 5'-CGGAGATGACCCTTTTG-3'  5'-GGTGCTGAGTATGTCGTGGA-3' |
| **IP_3_Rs**  Sense  Antisense | 5’- CTTCGCCAGGAACAGGAAACCCAC -3’  5’- CTCAGCTCGGGACGCGGCAG -3’ |
| **CXCR4**  Sense  Antisense | 5’- TAC-GCC-TTC-CTC-GGG-GCC-AA -3’  5’-CGG-AAG-AGT-GTC-CAC-CCC-GT -3’ |
| **RyRs**  Sense  Antisense | 5’- GTCACAGGATCCCAACGCAGCA -3’  5’- CCCGCGGTCATGCAGTCTGG -3’ |
